# Explanation

**Published:** 1865-06

**Authors:** 


Explanation—*Answer to the Canada Medical Journal.*—Dr. John C. Johnson's operation for exsection of ankle-joint, reported in the April number of this Journal, was made February 1862. Any one desirous of fixing the date more definitely will, no doubt, be supplied with the facts upon application; we are unable to supply them at present.

